# The resting behavior of malaria vectors in different ecological zones of Ghana and its implications for vector control

**DOI:** 10.1186/s13071-022-05355-y

**Published:** 2022-07-08

**Authors:** Akua Obeng Forson, Isaac A. Hinne, Shittu B. Dhikrullahi, Isaac Kwame Sraku, Abdul Rahim Mohammed, Simon K. Attah, Yaw Asare Afrane

**Affiliations:** 1 Department of Medical Laboratory Science, School of Biomedical and Allied Health Sciences, University of Ghana, Korle-Bu, Accra, Ghana; 2 Department of Medical Microbiology, University of Ghana Medical School, University of Ghana, Korle-Bu, Accra, Ghana

**Keywords:** Ghana, *Anopheles gambiae* sensu lato, Resting behavior, Insecticide resistance, Human blood index, Sporozoite rates

## Abstract

**Background:**

In sub-Saharan Africa there is widespread use of long-lasting insecticidal nets and indoor residual spraying to help control the densities of malaria vectors and decrease the incidence of malaria. This study was carried out to investigate the resting behavior, host preference and infection with *Plasmodium falciparum* of malaria vectors in Ghana in the context of the increasing insecticide resistance of malaria vectors in sub-Saharan Africa.

**Methods:**

Indoor and outdoor resting anopheline mosquitoes were sampled during the dry and rainy seasons in five sites in three ecological zones [Sahel savannah (Kpalsogo, Pagaza, Libga); coastal savannah (Anyakpor); and forest (Konongo)]. Polymerase chain reaction-based molecular diagnostics were used to determine speciation, genotypes for knockdown resistance mutations (L1014S and L1014F) and the G119S *ace1* mutation, specific host blood meal origins and sporozoite infection in the field-collected mosquitoes.

**Results:**

*Anopheles gambiae* sensu lato (s.l.) predominated (89.95%, *n* = 1718), followed by *Anopheles rufipes* (8.48%, *n* = 162) and *Anopheles funestus* s.l. (1.57%, *n* = 30). Sibling species of the *Anopheles gambiae* s.l. revealed *Anopheles coluzzii* accounted for 63% (95% confidence interval = 57.10–68.91) and 27% (95% confidence interval = 21.66–32.55) was *Anopheles gambiae s. s.*. The mean resting density of *An. gambiae* s.l. was higher outdoors (79.63%; 1368/1718) than indoors (20.37%; 350/1718) (Wilcoxon rank sum test,* Z* = − 4.815, *P *< 0.0001). The *kdr* west L1014F and the *ace1* mutation frequencies were higher in indoor resting *An. coluzzii* and *An. gambiae* in the Sahel savannah sites than in the forest and coastal savannah sites. Overall, the blood meal analyses revealed that a larger proportion of the malaria vectors preferred feeding on humans (70.2%) than on animals (29.8%) in all of the sites. Sporozoites were only detected in indoor resting *An. coluzzii* from the Sahel savannah (5.0%) and forest (2.5%) zones.

**Conclusions:**

This study reports high outdoor resting densities of *An. gambiae* and *An. coluzzii* with high *kdr* west mutation frequencies, and the presence of malaria vectors indoors despite the use of long-lasting insecticidal nets and indoor residual spraying. Continuous monitoring of changes in the resting behavior of mosquitoes and the implementation of complementary malaria control interventions that target outdoor resting *Anopheles* mosquitoes are necessary in Ghana.

**Graphical abstract:**

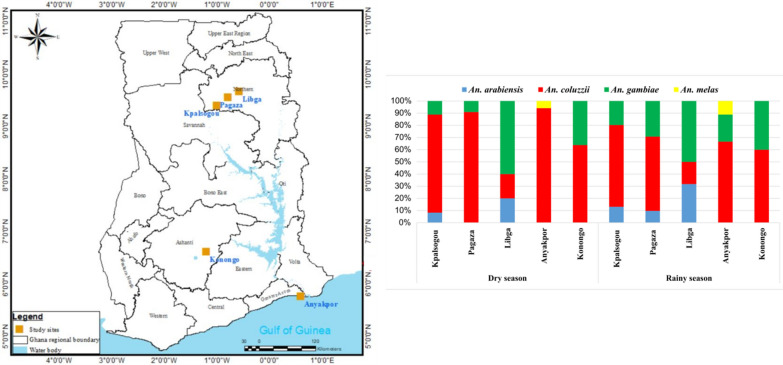

## Background

Malaria is a major public health problem in Africa, and was responsible for an estimated 241 million episodes and 624,000 deaths worldwide in 2020 [1]. In Ghana, malaria is responsible for more than 5.5 million infections and 37 deaths per 1000 population [2, 3] despite tremendous efforts to scale-up vector control interventions there, particularly in the use of long-lasting insecticidal nets (LLINs) and indoor residual spraying (IRS) [4, 5]. These anti-vector interventions led to a remarkable reduction in vector populations [6–9] and malaria transmission [10, 11] in the past. However, there are reports of a resurgence in malaria in many parts of sub-Saharan Africa [12, 13]. Some of the challenges associated with this resurgence include the emergence of insecticide resistance, behavioral modifications (shifts in the biting and resting behavior of vectors, from indoors to outdoors) and a shift in host species preference from humans to animals [7, 14–16].

These challenges have arisen as a consequence of adaptations of malaria vectors to the high use of insecticides for their control [17]. For instance, following the introduction of LLINs, there have been reports of a shift in the biting behavior of *Anopheles gambiae* and *Anopheles funestus* in Kenya [7, 18] and *Anopheles funestus* in Benin and Senegal [19, 20]. Whilst the long-term use of LLINs has increased the proportions of *Anopheles gambiae* and *Anopheles melas* feeding outdoors in Equatorial Guinea [14], in Tanzania, the long-term use of LLINs was reported to be associated with shifts in the outdoor resting rates of *Anopheles gambiae*, *Anopheles arabiensis* and *Anopheles funestus* [15, 21]. These behavioral changes, however, are not consistent, with some countries reporting high indoor resting densities of *An. gambiae* and *An. funestus* despite the long-term use of LLINs and IRS [22–24].

The widespread insecticide resistance of malaria vector populations in Africa is a major threat to current malaria control programmes there. Studies from Côte d’Ivoire [25], Togo [26, 27], Benin [28], Burkina Faso [29, 30], Cameroon [31, 32] and Kenya [33, 34] have reported high metabolic resistance and target site modifications in malaria vectors with respect to insecticides used against them. The acetylcholinesterase (*ace1*) target site mutation G119S, which enables resistance to organophosphates and carbamates, and the voltage-gated sodium channel knockdown resistance gene (*kdr*), which plays a major role in resistance to pyrethroids, are the most common and important target site mechanisms of mosquito vectors in Ghana [35–37].

The primary malaria vectors in Ghana are members of the *Anopheles gambiae* sensu lato (s.l.) species complex [*Anopheles gambiae* sensu stricto (s.s.), *Anopheles arabiensis*, *Anopheles coluzzii* and *Anopheles melas*] and *Anopheles funestus* s.s. [38–41]. In view of the resurgence of malaria transmission in Africa [42, 43], which is of increasing concern, there is a need to improve control intervention strategies through a better understanding of vector resting and feeding behavior in different settings and under varying seasonal patterns. This is crucial for the success of current vector control tools, and could provide a guide for improved efforts for the control of malaria in endemic regions.

The objective of this study was to investigate the resting behavior, species composition, insecticide resistance status and *Plasmodium falciparum* infections of malaria vectors in three ecological zones of Ghana (the coastal savannah zone in the south, the forest zone in the center, and the Sahel savannah zone in the north of the country). These ecological zones have varying climatic and other environmental conditions that are suitable for the propagation of *Anopheles* mosquitoes and malaria parasites [44]. The coastal savannah and forest zones have a bimodal rainfall pattern, allowing for two peaks of malaria transmission, while the Sahel savannah zone has a unimodal rainfall pattern leading to seasonal malaria transmission. The results of this study may improve our understanding of the impacts of current malaria control programmes on malaria vector populations, and their effects on mosquito resting behavior.

## Methods

### Study sites

**Fig. 1:**
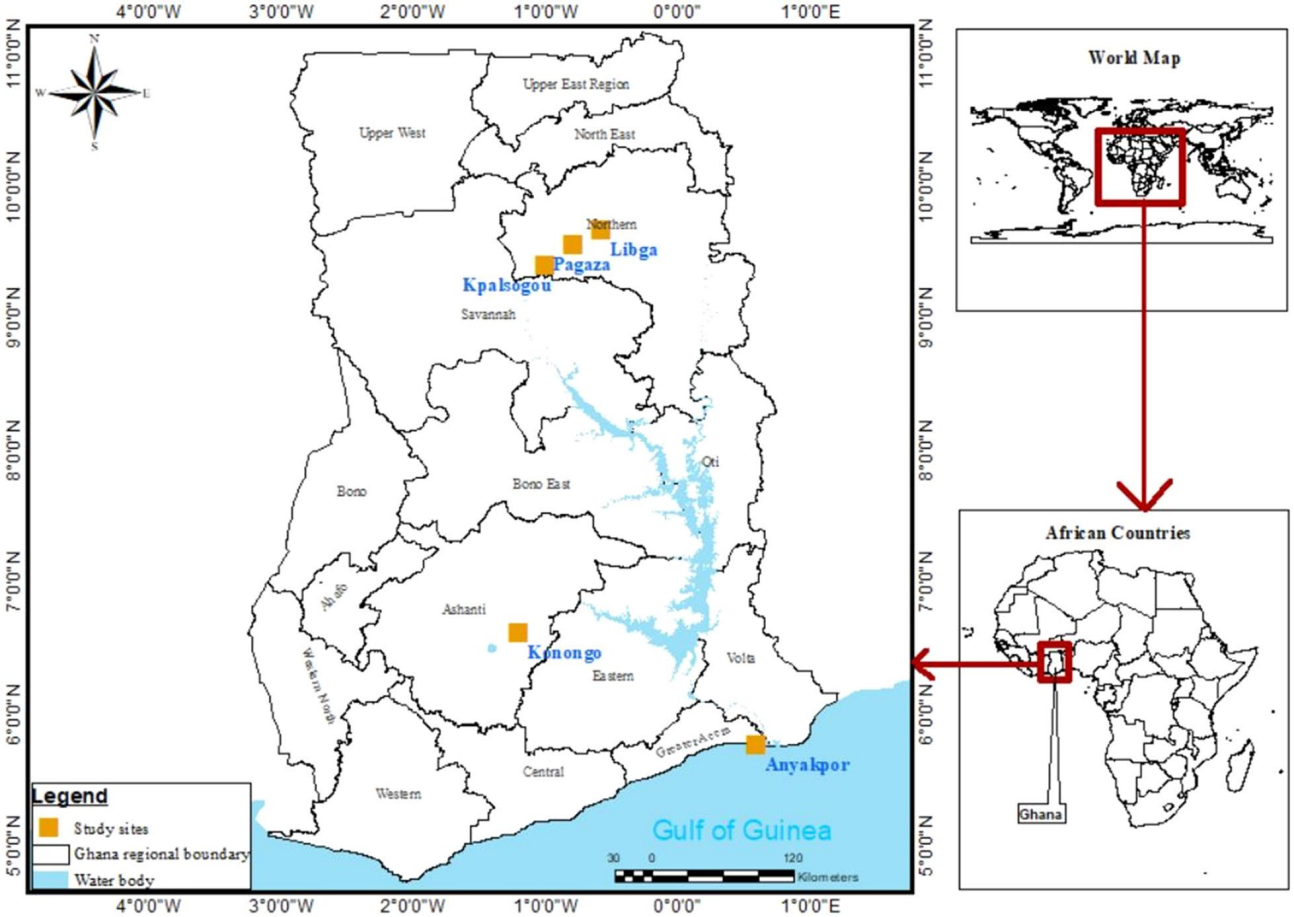
Map of Ghana showing the locations of the study sites

This study was carried out in five sites in three ecological zones of Ghana: Anyakpor in the coastal savannah zone; Dwease in the forest zone; and Kpalsogou, Libga and Pagaza in the Sahel savannah zone (Fig. 1).

Anyakpor (5°46′51.96"N, 0°35′12.84"E) is a village in the coastal savannah zone, about 5 km west of Ada Foah in southern Ghana. The coastal savannah has a tropical savannah climate, with average annual precipitation of 787 mm. Dwease (6°32′3.05"N, 1°14′42.22"W) is a village near Konongo, in the Asante-Akim Central District in the middle of Ghana, and is located in the forest zone. The forest zone has a tropical rainforest climate, with average annual precipitation of 1399.5 mm. Both the coastal savannah and forest zones generally have a bimodal pattern of rainfall, with the long rainy season from March to June and the short rainy season from October to November; their mean annual temperature is 26.5 °C. The sites in the Sahel savannah ecological zone were Kpalsogou (9°33′45.2"N, 1°01′54.6"W), a village in Kumbungu District in the northern region; Pagaza (9°22′33.34"N, 0°42′29.67"W) in the Tamale metropolitan area; and Libga (9°35′32.26"N, 0°50′48.8"W), a village in the Savelugu-Nanton Municipal District. These sites have a unimodal rainfall pattern from May to November and a mean annual temperature of 28 °C (the maximum recorded temperature is presently 42 °C).

### Mosquito collection

Mosquitoes were sampled in May 2019 during the rainy season at the sites in the coastal savannah and forest zones (Anyakpor and Konongo, respectively), in September 2019 at the sites in the Sahel savannah zone (Pagaza, Libga, and Kpalsogou), and in the dry season from February to March 2019 at all the study sites. Sixteen houses were randomly selected in each study site, and four of these houses were sampled for mosquitoes on each sampling night. Sampling was done over 4 days during both the dry and rainy seasons. The global positioning system coordinates of each site were determined and recorded.

Malaria vectors resting indoors were sampled from 0500 to 0700 hours using pyrethrum spray catches [45]. A Prokopack aspirator (John W. Hock, Gainesville, FL) was used from 0500 to 0700 hours to collect mosquitoes resting indoors or outdoors [46]. For indoor collection, mosquitoes resting on walls, under roofs, on ceilings, and under beds were systematically aspirated. Outdoor sampling points included kitchens, granaries, places where animals rested and locations where humans rested in the evening. Additionally, outdoor resting mosquitoes were collected from pit shelters constructed 10 m from selected houses according to Muirhead-Thomson’s method [47]. Resting mosquitoes were collected from 0600 to 0700 hours from cavities in the pit shelters by using hand-held mouth aspirators.

### Morphological identification

All of the mosquitoes caught were counted and anopheline mosquitoes were sorted morphologically according to the identification keys of Gillies and Coetzee [48]. Sampled mosquitoes were further classified according to abdominal status as unfed, freshly fed, half-gravid and gravid. Mosquitoes collected using each collection method were stored in separately labeled vials in 95% ethanol. Samples were stored at the insectary of the Department of Medical Microbiology, University of Ghana Medical School, Accra, Ghana until required for further processing.

### Sibling species discrimination

Sibling species of the *An. gambiae* s.l. species complex were distinguished using the protocols of Scott et al. [49] and Fanello et al. [50]. Template DNA was extracted from one leg of each mosquito and placed directly into the polymerase chain reaction (PCR) Master Mix for amplification.

### Detection of sporozoites

The head and thorax of mosquitoes in pooled samples (10 mosquitoes in each pool) were used to detect the presence of *P. falciparum* sporozoites by PCR as described by Echeverry et al. [51].

### Detection of blood meal sources

The abdomens of the blood-fed *Anopheles* mosquitoes were cut into transverse sections. Genomic DNA was extracted from the mosquito abdomens using the ZR DNA MicroPrep kit (Zymo Research, CA) following the manufacturer’s instructions. One universal reverse primer and five animal-specific (human, cow, goat, pig, and dog) forward primers were used for amplification of the mitochondrial cytochrome b gene to test for specific host blood meal origin using conventional PCR [52]. Positive controls were included for each host in the PCR analyses, and laboratory-reared unfed *An. gambiae* were used as the negative control.

### Genotyping for *kdr* and *ace1* mutations

For *kdr* mutation genotyping, DNA was extracted from mosquito legs using the ZR DNA MicroPrep kit (Zymo Research) following the manufacturer’s instructions. Standard PCR assays for the L1014F *kdr* allele were used to test for the presence of the *kdr* gene using a modification of the protocol described by Ahadji-Dabla et al. [53]. The G119S mutation of the *ace1* gene was assessed using the PCR protocol described by Weill et al. [54].

### Data analysis

Densities of resting anopheline mosquitoes were calculated as the number of female mosquitoes/trap per night for each trapping method. The Mann–Whitney* U*-test was used to compare malaria vector density between indoor and outdoor locations. The chi-square test was used to examine the differences in seasonal abundance and malaria vector species composition between resting locations (indoors and outdoors).

Human blood index (HBI) was calculated as the proportion of blood-fed mosquito samples that had fed on humans relative to the total tested for blood meal origin. The sporozoite infection rate, expressed as the proportion of mosquitoes positive for *Plasmodium* sporozoites, was calculated by dividing the number of sporozoite-positive mosquitoes by the total number of mosquitoes assayed.

The *kdr* L1014F and *ace1* G119S mutation frequencies were calculated according to the following formula [53]:

$$\mathrm{F }=\frac{2 \left(\mathrm{Homozygote \ Resistant}\right)+\mathrm{Heterozygote \ Resistant}}{2\left(\mathrm{total \ number \ of \ specimen \ analyzed}\right)}$$

Sporozoite infection rates were calculated for the pooled samples (10 samples per pool) of mosquitoes by using the formula given by Gu [55]:

$$S=\frac{\mathrm{No. \ of \ positive \ pools}}{\mathrm{No. \ of \ pools }\times \mathrm{ Maximum \ pool \ size}} \times 100{\%}$$

The maximum likelihood estimate (MLE) of the pooled mosquitoes was determined using the frequentist MLE model in R [56].

## Results

### Indoor and outdoor resting densities of female *Anopheles* mosquitoes

A total of 4810 mosquitoes belonging to four genera were collected during the sampling period. Of these, 1910 (39.71%) belonged to the genus *Anopheles*, 2814 (58.50%) to *Culex*, 82 (1.70%) to *Aedes*, and four (0.08%) to *Mansonia*. The 1910 *Anopheles* mosquitoes comprised 1718 (89.95%) *Anopheles gambiae* s.l., 162 (8.48%) *Anopheles* *rufipes*, and 30 (1.57%) *Anopheles* *funestus*. Overall, 81.57% (1558/1910) of the *Anopheles* mosquitoes caught at the different sites had been resting outdoors and 18.43% (352/1910) indoors (Wilcoxon rank sum test, *Z* =  − 4.970, *P* < 0.0001). The mean resting density of *An. gambiae* s.l. was higher outdoors (79.63%; 1368/1718) than indoors (20.37%; 350/1718) (Wilcoxon rank sum test, *Z* =  − 4.815, *P* < 0.0001). More *An. funestus* were resting outdoors 93.33%; 28/30) than indoors (6.67%; 2/30) (Wilcoxon rank sum test, *Z* = − 2.039, *P* < 0.0001). In addition, all (100%) of the *An. rufipes* caught in this study were resting outdoors.

In Kpalsogou, Pagaza and Libga (Sahel savannah zone), a total of 1372, 104 and 76 female anopheline mosquitoes, respectively, were caught, while in Anyakpor (coastal savannah zone) and Konongo (forest zone), a total of 52 and 114, respectively, were caught (Table 1). Out of the 1372 *An. gambiae* s.l. collected in Kpalsogou, 13.41% {184/1372 [95% confidence interval (CI) = 11.68–16.24]} were resting indoors and 86.59% [1188/1372 (95% CI = 84.64–88.32)] outdoors (Table 1). All of the *An. funestus* [100% (22/22)] and *An. rufipes* [100% (158/158)] were caught resting outdoors in Kpalsogou. In Pagaza and Libga, 7.7% [8/104 (95% CI = 3.62–15.04)] and 50.0% [38/76 (95% CI = 39.03–60.10)] of *An. gambiae* s.l. were caught resting indoors, respectively, and 92.3% [96/104 (95% CI = 84.96–96.38)] and 50.0% [38/76 (95% CI = 39.03–60.10)] outdoors, respectively. More *An. gambiae* s.l. were caught resting indoors in Anyakpor [84.6% (44/52) (95% CI = 71.37–92.66)] and Konongo [66.7% (76/114) (95% CI = 57.14–75.05] than outdoors [15.4% (8/52) (95% CI = 7.34–28.63) and 33.3% (38/114) (95% CI = 24.95–42.86), respectively].

**Table 1:** Resting densities of mosquitoes collected from different sites in three ecological zones of Ghana

Site	Mosquito species	Indoors [no. (%)]	Outdoors [no. (%)]	Total (no.)
Kpalsogou (Sahel savannah zone)	*Anopheles gambiae* s.l.	184 (13.41)	1188 (86.59)	1372
	*Anopheles funestus* s.l.	0	22 (100)	22
	*Anopheles rufipes*	0	158 (100)	158
Pagaza (Sahel savannah zone)	*An. gambiae* s.l.	8 (7.69)	96 (92.31)	104
	*An. funestus* s.l.	0	0	0
	*An. rufipes*	0	0	0
Libga (Sahel savannah zone)	*An. gambiae* s.l.	38 (50)	38 (50)	76
	*An. funestus* s.l.	2 (50)	2 (50)	4
	*An. rufipes*	0	0	0
Anyakpor (Coastal savannah zone)	*An. gambiae* s.l.	44 (84.62)	8 (15.38)	52
	*An. funestus* s.l.	0	0	0
	*An. rufipes*	0	4 (100)	4
Konongo (Forest zone)	*An. gambiae* s.l.	76 (66.67)	38 (33.33)	114
	*An. funestus* s.l.	0	4 (100)	4
	*An. rufipes*	0	0	0
Total	*An. gambiae* s.l.	350 (20.37)	1368 (79.63)	1718
	*An. funestus* s.l.	2 (6.67)	28 (93.33)	30
	*An. rufipes*	0	162 (100)	162

*s.l.* Sensu lato

### Seasonal densities of *Anopheles* mosquitoes from different sites

More anopheline mosquitoes [1214 (63.56%)] were sampled in the dry season than in the rainy season [696 (36.44%); Wilcoxon rank sum test, *Z* = − 1.503, *P* = 0.1329; Table 2]. In the Sahel savannah zone (Kpalsogou, Pagaza and Libga), a total of 1054 (67.91%), 10 (9.62%), 32 (45.5%) vs. 498 (32.1%), 48 (54.5%) and 94 (90.38%) female *Anopheles* mosquitoes were caught in the dry and rainy seasons, respectively. In Konongo (the forest zone), more *Anopheles* were caught in the dry season [88.14% (104/118)] than in the rainy season [11.86% (14/118)], while in Anyakpor (coastal savannah zone), more *Anopheles* were collected in the rainy season [75% (42/56)] than in the dry season [25% (14/56)].

**Table 2:** Total numbers of mosquitoes collected from different sites in three ecological zones of Ghana during the dry and rainy seasons

Season	Location	*Anopheles gambiae* s.l.	*Anopheles funestus* s.l.	*Anopheles rufipes*	Total
Dry	Kpalsogou	910	0	144	1054
	Pagaza	10	0	0	10
	Libga	32	0	0	32
	Anyakpor	14	0	0	14
	Konongo	104	0	0	104
	Subtotal (%)	1070 (62.3)	0	144 (88.9)	1214
Rainy	Kpalsogou	462	22	14	498
	Pagaza	94	0	0	94
	Libga	44	4	0	48
	Anyakpor	38	0	4	42
	Konongo	10	4	0	14
	Subtotal (%)	648 (37.7)	30 (100)	18 (11.1)	696
Total		1718 (100)	30 (100)	162 (100)	1910

In all, the abundance of *An. gambiae* s.l. was 62.28% [1070/1718 (95% CI = 59.94–64.57)] in the dry season and 37.72% [648/1718 (95% CI = 35.43–40.06)] in the rainy season (Table 2). *Anopheles rufipes* was also more abundant in the dry season [88.89%, 144/162 (95% CI = 79.48–94.48)] than in the rainy season [11.11%, 18/162 (95% CI = 6.70–17.24)]. *Anopheles funestus* was only caught during the rainy season at Kpalsogou, Libga and Anyakpor. No *An. funestus* were caught in the dry season at any of the study sites.

### *Anopheles gambiae* sibling species composition

A sub-sample of 538 *An. gambiae* s.l. from all the study sites were identified to sibling species. Overall, *An. coluzzii* accounted for 63% (95% CI = 57.1–68.9), followed by *An. gambiae* s.s. [hereafter (*An. gambiae*)], which accounted for 27% (95% CI = 21.7–32.6), then *An. arabiensis*, which accounted for 9% (95% CI = 6.2–13.6) and *An. melas*, which accounted for 1% (95% CI = 0.1–3.0). A total of 194 of 1362 (12%) of *An. gambiae* s.l. from Kpalsogou were analyzed, of which 72% [140/194 (95% CI = 61.9–50.6)] were *An. coluzzii*, 17% [32/194 (95% CI = 10.1–25.7)] were *An. gambiae* and 11% [22/194 (95% CI = 6.1–19.8)] were *An. arabiensis*. Out of the 104 *An. gambiae* s.l. analyzed from Pagaza, 67% [70/104 (95% CI = 52.8–79.3)] were *An. coluzzii*, 25% [26/104 (95% CI = 14.5–39.3)] were *An. gambiae* and 8% [8/104 (95% CI = 2.5–19.4)] were *An. arabiensis*. Out of the 74 *An. gambiae* s.l. analyzed from Libga, 54% [40/74 (95% CI = 37.1–70.2)] were *An. gambiae*, 27% [20/74 (95% CI = 14.4–44.4)] were *An. arabiensis* and 19% [14/74 (95% CI = 8.6–35.7)] were *An. coluzzii*. For Anyakpor (the coastal zone), out of the 52 *An. gambiae* s.l. analyzed, 84% [44/52 (95% CI = 64.3–94.9)] were *An. coluzzii* and the rest were *An. gambiae* [8% (4/52); 95% CI = 1.3–26.6] and *An. melas* [8% (4/52); 95% CI = 0.1–2.9]. In contrast, only *An. coluzzii* [63% (72/114); 95% CI = 49.0–75.2] and *An. gambiae* [37% (42/114); 95% CI = 24.7–50.7] were identified from the forest zone.

**Fig. 2:**
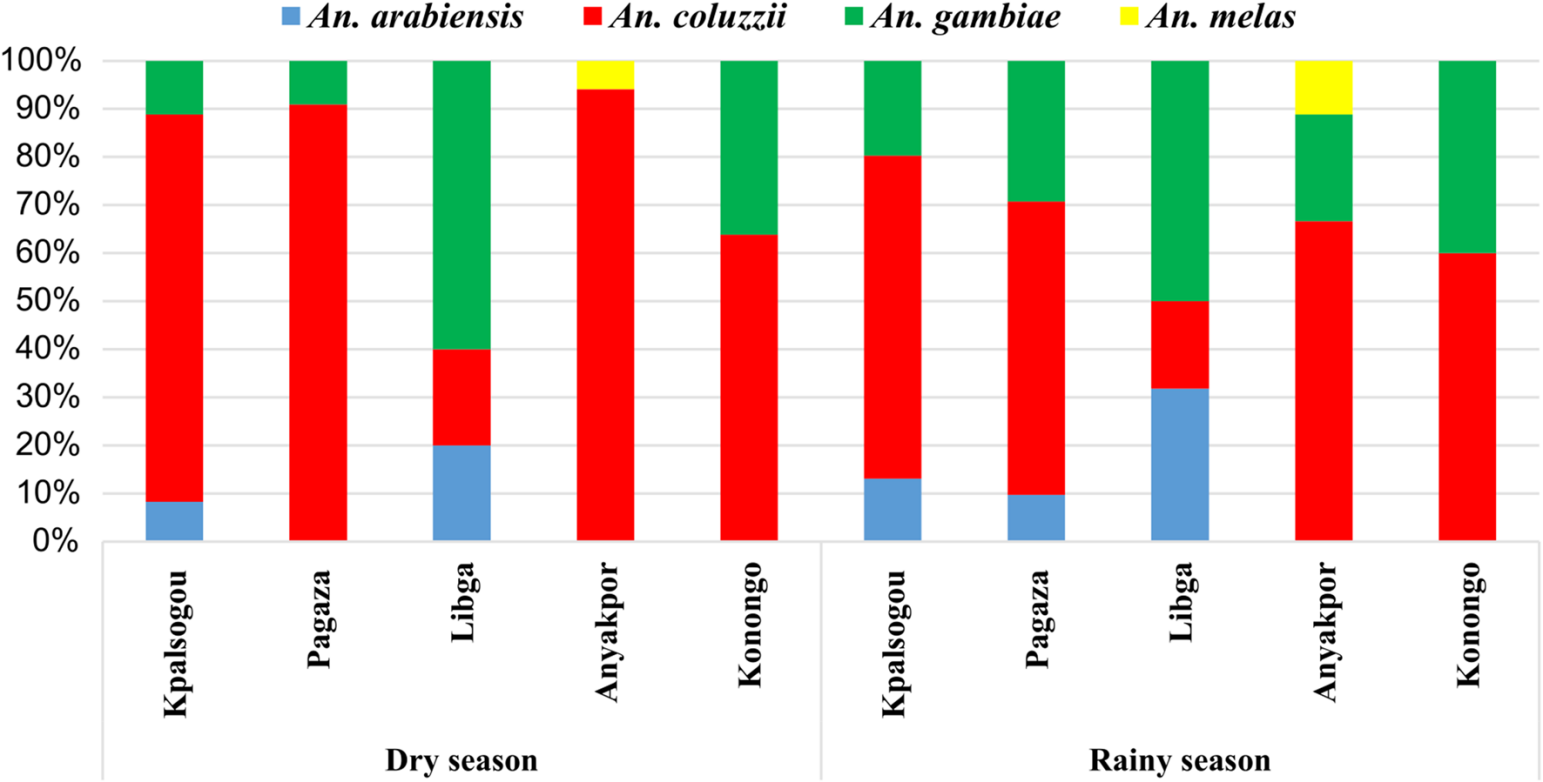
Seasonal composition of resting *Anopheles* (*An.*) species in the dry season and rainy season in different sites of three ecological zones of Ghana

The seasonal composition of the two malaria vectors *An. coluzzii* and *An. gambiae* varied at the different sites. In the Sahel savannah zone, the percentage of *An. coluzzii* was higher in the dry season (81% and 91%) than in the rainy season (67% and 61%) for Kpalsogou and Pagaza, respectively (Fig. 2). In contrast, the percentage of *An. gambiae* was higher in the dry season (60%) than in the rainy season (50%) in Ligba. In Anyakpor and Konongo, higher densities of *An. coluzzii* were detected in the dry season (94% and 64%, respectively) compared to the rainy season (66% and 60%, respectively).

The frequencies of *Anopheles* resting indoors and outdoors at the different sites varied by season. In the dry season, in Kpalsogou and Pagaza, more *Anopheles* were caught resting outdoors (32% and 3.7%, respectively) than indoors (14% and 0.7%, respectively) (Table 3). At Konongo, more *Anopheles* mosquitoes were caught resting indoors (30.9%) than outdoors (4.4%) in the dry season. In the rainy season, the proportions of *Anopheles* mosquitoes resting indoors and outdoors were similar for Kpalsogou (12.8% vs. 12.8%) and Libga (7.5% vs. 7.5%). However, densities of collected mosquitoes were higher indoors than outdoors at Anyakpor (12% vs. 3%) and Konongo (3.8% vs. 3%).

**Table 3:** Proportions of members of the *Anopheles gambiae* species complex resting indoors and outdoors at different sites in three ecological zones of Ghana

Species	Locations										Total (%)
	Kpalsogou		Pagaza		Libga		Anyakpor		Konongo		
	Indoor	Outdoor	Indoor	Outdoor	Indoor	Outdoor	Indoor	Outdoor	Indoor	Outdoor	
Dry season											
*Anopheles arabiensis*	0	10	0	2	0	10	0	0	0	0	22 (8.1)
*Anopheles coluzzii*	36	56	2	6	4	4	2	2	54	8	174 (64)
*Anopheles gambiae*	2	22	0	2	6	10	0	0	30	4	76 (27.9)
*Anopheles melas*	0	0	0	0	0	0	0	0	0	0	0
Total (%)	38 (14)	88 (32.4)	2 (0.7)	10 (3.7)	10 (3.7)	24 (8.8)	2 (0.73)	2 (0.7)	84 (30.9)	12 (4.4)	272
Rainy season											
*An. arabiensis*	6	6	0	6	6	4	0	0	0	0	28 (10.5)
*An. coluzzii*	22	26	18	44	2	4	30	10	6	4	166 (62.4)
*An. gambiae*	6	2	2	22	12	12	0	4	4	4	68 (25.6)
*An. melas*	0	0	0	0	0	0	2	2	0	0	4 (1.5)
Total (%)	34 (12.8)	34 (12.8)	20 (7.5)	72 (27.1)	20 (7.5)	20 (7.5)	32 (12.0)	16 (6.0)	10 (3.8)	8 (3.0)	266

### *kdr *resistance mutations of indoor resting compared to outdoor resting *An. gambiae*

A total of 538 *An. gambiae* s.l. samples were genotyped for the presence of the L1014S, L1014F and G119S* ace1* mutations. The L1014F allele of *kdr* was identified in 100% (538) of the samples, with the majority of mosquitoes being homozygous for the *kdr* allele (70.6%; 380/538). Overall, there was little difference between the *kdr* mutation frequencies of mosquitoes collected indoors and outdoors for all the study sites (Table 4). Furthermore, the *kdr* L1014S mutation was not detected in this study.

**Table 4:** Frequency of the *kdr* L1014F mutation in members of the *Anopheles gambiae* species complex at different sites in three ecological zones of Ghana

Site	Location	*An. arabiensis*				*An. coluzzii*				*An. gambiae*				*An. melas*			
		*Kdr*L1014				*Kdr*L1014				*Kdr*L1014				*Kdr*L1014			
		No. tested	RS	RR	F(*Kdr*)	No. tested	RS	RR	F(*Kdr*)	No. tested	RS	RR	F(*Kdr*)	No. tested	RS	RR	F(*Kdr*)
Kpalsogou	Indoor	6	0	6	1	58	12	46	0.9	8	0	8	1				
	Outdoor	16	4	12	0.9	82	26	56	0.8	24	4	20	0.9				
	Total	22				140				32							
Pagaza	Indoor	0	0	0	0	20	4	16	0.9	2	2	0	0.5				
	Outdoor	8	4	4	0.8	50	30	20	0.7	24	6	18	0.9				
	Total	8				70				26							
Libga	Indoor	6	2	4	0.8	6	0	6	1	18	6	12	0.8				
	Outdoor	14	4	10	0.9	8	0	8	1	22	2	20	1.0				
	Total	20				14				40							
Anyakpor	Indoor					32	4	28	0.9	0	0	0	0	2	2	0	0.5
	Outdoor					12	0	12	1	4	2	2	0.8	2	0	2	1
	Total					44				4				4			
Konongo	Indoor					60	24	36	0.8	34	16	18	0.8				
	Outdoor					12	0	12	1.0	8	4	4	0.8				
	Total					72				42							

*RR* Homozygote resistant, *RS* heterozygote resistant, *F* frequency

*Anopheles arabiensis* collected outdoors at Pagaza and Libga had higher *kdr* mutation frequencies (0.75 and 0.86, respectively) than those collected indoors (0 and 0.8, respectively). However, in Kpalsogou, *An. arabiensis* collected indoors had a higher *kdr* mutation frequency (1) than those collected outdoors (0.9). *An. coluzzii* resting indoors caught in Kpalsogou and Pagaza had higher *kdr* mutation frequencies (both 0.9) than those resting outdoors (0.8 and 0.7, respectively). In Pagaza and Libga, *An. gambiae* caught outdoors had higher *kdr* mutation frequencies (0.9 and 1.0, respectively) than those resting indoors (0.5 and 0.8, respectively).

### G119S *ace1* mutations in indoor and outdoor resting malaria vectors

The G119S *ace1* mutation was detected in 79.9% (215/538) of the mosquitoes tested (Table 5). All the mosquitoes with the resistant allele were heterozygous for this mutation. Overall, similar *ace1* mutation frequencies were detected for indoor and outdoor resting mosquitoes in the Sahel savannah zone (0.8 vs. 0.8). In the forest zone, the frequency of *ace1* mutations was slightly higher in mosquitoes resting indoors (0.9) than in those resting outdoors (0.8), but was higher in mosquitoes resting outdoors (1) than those resting indoors (0.8) in the coastal zone. The frequency of the *ace1* mutation was similar for *An. coluzzii* collected indoors and outdoors in Kpalsogou (0.4 vs. 0.4), Libga (0.5 vs. 0.5) and Pagaza (0.4 vs. 0.4) (Table 5). In Kpalsogou and Pagaza (Sahel savannah sites), the *ace1* mutation frequency was higher for *An. gambiae* resting indoors (0.5 and 0.5, respectively) than outdoors (0.4 and 0.4, respectively).

**Table 5:** Frequencies of *ace1* mutation of *Anopheles gambiae* s.l. at different sites in three ecological zones of Ghana

Site	Species	Total No. Tested	No. Tested		Genotype			F (*ace*-1)
					GS	GG	SS	
					No. (%)	No. (%)		
Kpalsogou	*An. arabiensis*	22	6	Indoor	4 (18.2)	2 (9.1)	0	0.3
			16	Outdoor	6 (27.3)	10 (45.5)	0	0.2
	*An. coluzzii*	140	58	Indoor	44 (31.4)	14 (10)	0	0.4
			82	Outdoor	64 (45.7)	18(12.9)	0	0.4
	*An. gambiae*	32	8	Indoor	4 (25)	0	0	0.5
			24	Outdoor	18 (56.3)	6 (18.8)	0	0.4
Pagaza	*An. arabiensis*	8	0	Indoor	0	0	0	0.0
			8	Outdoor	8 (100)	0	0	0.5
	*An. coluzzii*	70	20	Indoor	14 (20)	6 (8.6)	0	0.4
			50	Outdoor	42 (60)	8 (11.4)	0	0.4
	*An. gambiae*	26	2	Indoor	2 (7.7)	0	0	0.5
			24	Outdoor	20 (76.9)	4 (15.4)	0	0.4
Libga	*An. arabiensis*	20	6	Indoor	2 (20)	4 (20)	0	0.2
			14	Outdoor	12 (60)	2 (10)	0	0.4
	*An. coluzzii*	14	6	Indoor	6 (42.9)	0	0	0.5
			8	Outdoor	8 (57.1)	0	0	0.5
	*An. gambiae*	40	18	Indoor	14 (35)	4 (10)	0	0.4
			22	Outdoor	16 (40)	6 (15)	0	0.4
Anyakpor	*An. coluzzii*	44	32	Indoor	24 (54.5)	8 (18.2)	0	0.4
			12	Outdoor	12 (27.3)	0	0	0.5
	*An. gambiae*	4	0	Indoor	0	0	0	0.0
			4	Outdoor	4 (100)	0	0	0.5
	*An. melas*	4	2	Indoor	2 (50)	0	0	0.5
			2	Outdoor	2 (50)	0	0	0.5
Konongo	*An. coluzzii*	72	60	Indoor	52 (72.2)	8 (11.1)	0	0.4
			12	Outdoor	10 (13.9)	2 (2.8)	0	0.4
	*An. gambiae*	42	34	Indoor	30 (71.4)	4(9.5)	0	0.4
			8	Outdoor	6 (14.3)	2 (4.8)	0	0.4

For abbreviations, see Table 3

### Blood meal sources

The 362 *An. gambiae* s.l. blood-fed mosquito specimens analyzed for blood meal origins had a HBI of 70.2% (254/362) across all sites. Overall, indoor resting mosquitoes had a slightly higher [73.1% (136/186)] HBI than outdoor [67% (118/176)] resting ones. In the savannah zone (Kpalsogou, Libga and Pagaza), the overall HBI of mosquitoes resting outdoors was higher [67.5% (52/77)] than that of indoor resting mosquitoes [32.5% (25/77)]. In Kpalsogou, the HBI for *An. arabiensis*, *An. coluzzii* and *An. gambiae* caught resting indoors and outdoors was 100%, 68.4% and 50% vs. 40%, 70% and 54.5%, respectively (Table 6). Only indoor and outdoor resting *An. coluzzii* were positive for human blood, with an HBI of 66.7% and 100%, respectively, in the coastal zone (Anyakpor). In Konongo (forest zone) the overall HBI was similar for indoor (84.7%) and outdoor (85.7%) resting mosquitoes. The HBI for *An. coluzzii* and *An. gambiae* caught resting indoors and outdoors was 92.3% and 75% vs. 88.2% and 100%, respectively, at the Konongo site.

**Table 6:** Blood meal origins of *Anopheles* mosquitoes collected indoors and outdoors at different sites in three ecological zones of Ghana

Site	Blood meal origins	*Anopheles arabiensis*		*Anopheles coluzzii*		*Anopheles gambiae*	
		Indoor	Outdoor	Indoor	Outdoor	Indoor	Outdoor
Kpalsogou	No. tested	2	10	8	60	4	22
	Human	2 (100)	4 (40)	26 (68.4)	42 (70)	2 (50)	12 (54.5)
	Goat	0	6 (60)	10 (26.5)	10 (16.7)	2 (50)	10 (45.5)
	Cow	0	0	2 (5.3)	4 (6.7)	0	0
	Dog	0	0	0	4 (6.7)	0	0
	HBI	100	40	68.4	70	50	54.5
	BBI	0	0	5.3	6.7	0	0
Pagaza	No. tested	0	6	16	8	2	8
	Human	0	4 (66.7)	8 (50)	14 (77.8)	2 (100)	6 (75)
	Goat	0	2 (33.3)	8 (50)	4 (22.2)	0	2 (25)
	HBI	0	66.7	50	77.8	100	75
	BBI	0	0	0	0	0	0
Libga	No. tested	6	10	2	8	12	16
	Human	4 (66.7)	8 (80)	2 (100)	8 (100)	4 (33.3)	6 (37.5)
	Goat	2 (33.3)	2 (20)	0	0	8 (66.7)	8 (50.0)
	Dog	0	0	0	0	0	2 (12.5)
	HBI	66.7	80	100	100	33.3	37.5
	BBI	0	0	0	0	0	00
Anyakpor	No. tested			12	1	0	2
	Human			8 (66.7)	2 (100)	0	0
	Dog			4 (33.3)	0	0	2 (100)
	HBI			66.7	100	0	0
	BBI			0	0	0	0
Konongo	No. tested			52	8	34	6
	Human			48 (92.3)	6 (75)	30 (88.2)	6 (100)
	Goat			4 (7.7)	0	4 (11.8)	0
	Pig			0	2 (25)	0	0
	Unidentified			3	0	0	0
	HBI			92.3	75	88.2	100
	BBI			0	0	0	0

*HBI* Human blood index, *BBI* bovine blood index

### Sporozoite infection rates

A total of 64 pools (each pool consisted of material from 10 mosquitoes) of the head and thorax of anopheline mosquitoes (18 pools for Kpalsogou, eight for Pagaza, 16 for Libga, eight for Anyakpor and 14 for Konongo) were tested for *P. falciparum* circumsporozoite protein (CSP). Four pools (two for Kpalsogou and two for Konongo) tested positive for *P. falciparum* CSP. The MLE prevalence for the pooled mosquitoes was 0.6 (95% CI = 0.2–1.5). The calculated sporozoite infection rate was higher for indoor resting mosquitoes from Kpalsogou (5.0%) and Konongo (2.5%) (Table 7). None of the mosquitoes collected outdoors in Kpalsogou, Pagaza, Libga, Anyakpor or Konongo were positive for *P. falciparum* CSP. CSP was only detected in *An. coluzzii* collected in the rainy season in Kpalsogou (2.5%) and in the dry season in Konongo (2.0%).

**Table 7:** Sporozoite infections detected in pooled *Anopheles gambiae* s.l. from different sites in three ecological zones of Ghana

	Location	*Anopheles arabiensis*		*Anopheles coluzzii*		*Anopheles gambiae*	
		Pools tested (no.)	Pf CSP +ve (no.)	Pools tested (no.)	Pf CSP +ve (no.)	Pools tested (no.)	Pf CSP +ve (no.)
Kpalsogou	Indoor	–	–	4	2 (5%)	–	–
	Outdoor	2	0	6	0	6	0
Pagaza	Indoor	–	–	–	–	4	0
	Outdoor	2	0	2	0	–	–
Libga	Indoor			4	0		
	Outdoor			12	0		
Anyakpor	Indoor			4	0	–	–
	Outdoor			0	0	2	0
Konongo	Indoor			8	2 (2.5%)	2	0
	Outdoor			2	0	2	0

*Pf CSP +ve * Positive for *Plasmodium falciparum* circumsporozoite protein

## Discussion

Behavioral diversification in vector populations in areas with widespread use of LLINs and IRS is a threat to the efficacy of vector control strategies [7, 8, 14–16, 57]. This study investigated the behavior of malaria vectors, specifically their resting and feeding choices, and their rates of infection with *Plasmodium falciparum* sporozoites, in the context of increasing insecticide resistance. Overall, this study revealed high outdoor resting densities of malaria vectors, but that these mosquitoes had lower sporozoite infection rates than those resting indoors. More mosquitoes with insecticide resistance mutations were found resting indoors than outdoors at the different sites in the three ecological zones of Ghana included in this study.

The use of LLINs and IRS in Kpalsogou, Libga and Pagaza (Sahel savannah zone) is driving more malaria vectors to rest outdoors, as observed for *An. coluzzii* and *An. gambiae* collected from different sites in a previous study in Ghana [58]. This could lead to an increase in malaria transmission outdoors since these main malaria vectors can bite unprotected humans outdoors and also rest outdoors to avoid contact with the insecticides that are used indoors [59, 60]. This behavioral change in malaria vector populations is detrimental to the efficacy of LLINS and IRS vector control strategies as these mainly target vectors resting indoors. A tendency for the malaria vectors (*An. coluzzii* and *An. gambiae*) to rest and feed outdoors due to the long-term use of LLINs has been reported from Equatorial Guinea [14] and Tanzania [15, 21].

Generally, more mosquitoes were collected during the rainy season than the dry season, apart from in Kpalsogou and Konongo. Kpalsogou has a dam for irrigation, and breeding habitats are created when the water held in the dam flows through the conduits to the farmland that it feeds. In Konongo, breeding habitats are found along a stream that flows through the village. However, both the dam and the stream flood during the rainy season, and mosquito larvae are washed away as a result. This might explain the low vector abundance found in Konongo and Kpalsogou during the rainy season.

One interesting observation was that, the frequencies of the *kdr* west mutation L1014F and the *ace1* mutation were higher in indoor resting *An. coluzzii* and *An. gambiae* in the Sahel savannah sites compared to the forest and coastal savannah sites. This may be because the increase in IRS and LLINs use in the Sahel savannah sites has selected for malaria vectors with *kdr* and *ace1* mutations. In addition, a behavioral change in populations of malaria vectors was observed in previous studies [31, 67–69], whereby those with low *kdr* mutation frequencies rested outdoors and thus avoided contact with the insecticides that were used indoors. Previous studies from Ghana [35, 36] have reported similar frequencies of *kdr* L1014F in *An. coluzzii* to that found in the present study. Similar to other studies carried out in Ghana [35, 36], no *kdr* east allele was detected in the present study. However, *kdr* east allele 1014S has been reported in *An. coluzzii*, *An. gambiae*, and *An. arabiensis* from Burkina Faso [61], and in both *An. coluzzii* and *An. gambiae* from Togo [27].

The blood meal analyses revealed that a large proportion of the malaria vectors preferred feeding on humans than on animals in almost all of the sites. This preference for human hosts and the higher proportions of outdoor resting *An. gambiae* and *An. coluzzii* are of great concern for malaria elimination efforts due the efficacy of these mosquitoes in transmitting malaria. A similar study to ours carried out by Orsborne et al. [62] in the coastal area of Ghana likewise reported that blood-fed mosquitoes caught indoors had higher HBI and a lower bovine blood index than those caught outdoors. Sporozoite infections were only found in indoor resting malaria vectors, collected during the wet season from Kpalsogou and during the dry season from Konongo. This indicates that malaria transmission may occur more indoors than outdoors [63]. However, in comparison to the results of the present study, higher sporozoite infections were found in *An. gambiae* and *An. coluzzii* resting outdoors in Kenya and Burkina Faso [22, 57, 64], and in *An. gambiae* sampled outdoors in northern Ghana [8].

Environmental conditions, including climate, have affected the distribution and species composition of malaria mosquitoes in the three main ecological zones of Ghana included in this study [39]. In the coastal savannah zone, *An. melas* thrive due to their tolerance of high salinity [65, 66], whilst in the Sahel savannah zone some *An*. *arabiensis* are present due to the dry conditions (low humidity) [67]. In Ghana as a whole, *An. coluzzii* and *An. gambiae* are the dominant species of* Anopheles*, but their distribution and abundance within the country are affected by environmental factors. Whilst *An. coluzzii* is dominant in the Sahel savannah zone, which is drier (less humid), in the forest zone, which has high rainfall and high humidity, *An. gambiae* is dominant [40, 68]. Thus climatic factors influence the distribution and abundance of the *An. gambiae* species complex in the various ecological zones of Ghana. The rapid deforestation of the Konongo area, which was not taken into account in this study, might have affected the climatic conditions there, which in turn may have allowed *An. coluzzii* to thrive in contrast to *An. gambiae* s.s. [69].

An important limitation of this study was not documenting the level of use of LLINs in each study site to better understand the changes in the resting behavior of the malaria vectors. However, the coverage of LLINs exceeds 70% in the study sites [70]

## Conclusions

This study revealed high densities of *An. coluzzii* and *An. gambiae* with low genotypic insecticide resistance resting outdoors compared to indoors, which may have been triggered by current insecticide-based indoor interventions. This behavioral change in mosquito vectors could promote the outdoor transmission of malaria, since current control strategies mainly target indoor resting malaria vectors. There is a need for the further screening of resistance mutations in populations of these vectors to improve management strategies for their control. In addition, continuous monitoring of vector behavior through surveillance programmes is recommended, and there is a need for complementary malaria control interventions to control outdoor resting mosquitoes.

## Data Availability

The datasets used and/or analyzed during the current study are available from the corresponding author on reasonable request.

## Abbreviations

CSP: Circumsporozoite protein
HBI: Human blood index
IRS: Indoor residual spraying
KDR: Knockdown resistant gene
LLINs: Long-lasting insecticidal nets
MLE: Maximum likelihood estimate
PCR: Polymerase chain reaction
